# Persistent lymphocyte reduction and interleukin-6 levels are independently associated with death in patients with COVID-19

**DOI:** 10.1007/s10238-023-01114-0

**Published:** 2023-06-13

**Authors:** You Xu, Nianci Wang, Xiao Shen, Xu Liu, Han Liu, Ying Liu

**Affiliations:** Department of Critical Care Medicine, Nanjing First Hospital, Nanjing Medical University, Nanjing, 210006 Jiangsu People’s Republic of China

**Keywords:** Novel coronavirus infection, Pneumonia, Interleukin-6, Lymphocyte count, Prognosis

## Abstract

To investigate the value of the peripheral blood lymphocyte count (LYM) combined with interleukin-6 (IL-6) in predicting disease severity and prognosis in patients with severe acute respiratory syndrome coronavirus 2 (SARS-CoV-2) pneumonia. This was a prospective observational cohort study. A total of 109 patients with SARS-CoV-2 pneumonia who were admitted to Nanjing First Hospital from December 2022 to January 2023 were enrolled. The patients were divided into two groups based on disease severity: severe (46 patients) and critically ill (63 patients). The clinical data of all patients were collected. The clinical characteristics, sequential organ failure assessment (SOFA) score, peripheral blood lymphocyte count, IL-6 level and other laboratory test results were compared between the two groups. A receiver operating characteristic (ROC) curve was plotted to evaluate the predictive value of each index for SARS-CoV-2 pneumonia severity; patients were regrouped using the optimal cut-off value of the ROC curve, and the relationship between different LYM and IL-6 levels and the prognosis of patients was analysed. Kaplan‒Meier survival curve analysis was performed; in the different LYM and IL-6 groups, the patients were regrouped based on whether thymosin was used, and the effect of thymosin on patient prognosis was compared between the groups. Patients in the critically ill group were significantly older than patients in the severe group (age: 78 ± 8 vs. 71 ± 17, t = 2.982, *P* < 0.05), and the proportion of patients with hypertension, diabetes and cerebrovascular disease was significantly higher in the critically ill group than in the severe group (69.8% vs. 45.7%, 38.1% vs. 17.4%, 36.5% vs. 13.0%; χ^2^ values, 6.462, 5.495, 7.496, respectively, all *P* < 0.05). Compared with the severe group, the critically ill group had a higher SOFA score on admission (score: 5.4 ± 3.0 vs. 1.9 ± 1.5, t = 24.269, *P* < 0.05); IL-6 and procalcitonin (PCT) in the critically ill group were significantly higher than those in the severe group on the first day of admission [288.4 (191.4, 412.9) vs. 513.0 (288.2, 857.4), 0.4 (0.1, 3.2) vs. 0.1 (0.05, 0.2); Z values, 4.000, 4.456, both *P* < 0.05]. The lymphocyte count continued to decline, and the lymphocyte count on the 5th day (LYM-5d) was still low (0.6 ± 0.4 vs. 1.0 ± 0.4, t = 4.515, both *P* < 0.05), with statistically significant differences between the two groups. ROC curve analysis indicated that LYM-5d, IL-6 and LYM-5d + IL-6 all had value for predicting SARS-CoV-2 pneumonia severity; the areas under the curve (AUCs) were 0.766, 0.725, and 0.817, respectively, and the 95% confidence intervals (95% CI) were 0.676–0.856, 0.631–0.819, and 0.737–0.897, respectively. The optimal cut-off values for LYM-5d and IL-6 were 0.7 × 10^9^/L and 416.4 pg/ml, respectively. LYM-5d + IL-6 had the greatest value in predicting disease severity, and LYM-5d had higher sensitivity and specificity in predicting SARS-CoV-2 pneumonia severity. Regrouping was performed based on the optimal cut-off values for LYM-5d and IL-6. Comparing the IL-6 ≥ 416.4 pg/ml and LYM-5d < 0.7 × 10^9^/L group with the other group, i.e., patients in the non–low-LYM-5d and high-IL-6 group, patients in the low-LYM-5d and high-IL-6 group had a higher 28-day mortality rate (71.9% vs. 29.9%, χ^2^ value 16.352, *P* < 0.05) and a longer hospital stay, intensive care unit (ICU) stay and mechanical ventilation time (days: 13.7 ± 6.3 vs. 8.4 ± 4.3, 9.0 (7.0, 11.5) vs. 7.5 (4.0, 9.5), 8.0 (6.0, 10.0) vs. 6.0 (3.3, 8.5); t/Z values, 11.657, 2.113, 2.553, respectively, all *P* < 0.05), as well as a higher incidence of secondary bacterial infection during the disease course (75.0% vs. 41.6%, χ^2^ value 10.120, *P* < 0.05). Kaplan‒Meier survival analysis indicated that the median survival time of patients in the low LYM-5d and high-IL-6 group was significantly shorter than that of patients in the non-low LYM-5d and high-IL-6 group (14.5 ± 1.8 d vs. 22.2 ± 1.1 d, Z value 18.086, *P* < 0.05). There was no significant difference in the curative effect between the thymosin group and the nonthymosin group. LYM and IL-6 levels are closely related to SARS-CoV-2 pneumonia severity. The prognosis for patients with IL-6 ≥ 416.4 pg/ml at admission and a lymphocyte count < 0.7 × 10 9/L on the 5th day is poor.

## Introduction

Severe acute respiratory syndrome coronavirus 2 (SARS-CoV-2) variant B.1.1.529 (Omicron) was first detected in South Africa on 25 November 2021 [1] and was listed by the World Health Organization (WHO) as a variant of concern. A variety of SARS-CoV-2 strains have become predominant worldwide [2]. Since 2022, multiple Omicron variants have caused outbreaks in China, including the BA.1 variant in Tianjin and the BA.2 variant in Shanghai. The main variant strains recently prevalent in China were the BA.5.2 and BF.7 sublineages of BA.5 [3]. Novel coronavirus disease (COVID-19) most often affects the respiratory system, leading to pneumonia. The early identification of infected patients and timely treatment can reduce the mortality rate and improve patient prognosis. Recent studies have shown that early variants of Omicron cause less severe symptoms [4], but data from France indicated that the mortality rates of severe COVID-19 caused by the Omicron and Delta variants were similar and that there was no statistically significant difference in the mortality rate of different variants of Omicron (BA.1/BA.1.1 and BA.2) [5]. The BA.5.2 variant and BF.7 variant that were recently prominent in China have been associated with many severe and fatal cases.

The pathogenesis of COVID-19 involves excessive activation of the body's immune system. Interleukin-6 (IL-6), as a key factor in the body's proinflammatory response, can activate the JAK-STAT pathway and induce an inflammatory response, possibly a cytokine storm, which is a key factor for the development of acute respiratory distress syndrome (ARDS) and other extrapulmonary organ injuries. Peripheral blood IL-6 levels are an independent risk factor for predicting disease progression and death. A study showed higher levels of IL-6 in deceased patients than in surviving patients [6], and IL-6 levels > 32.1 ng/L have been shown to predict more serious complications involving extrapulmonary organs [7]. In the late stage of infection, some patients progress to an immunosuppressed state. Studies have reported that when the lymphocyte count remains lower than 1.0 × 10^9^/L (3 d–4 d), patients are likely to be in an immunosuppressed state [8] and that a lymphocyte count of < 500/μl suggests a poor prognosis [3, 9]. Given the many people with congenital or acquired immunosuppression worldwide, there is an association between immunosuppression and the development of highly contagious or more pathogenic variants of SARS-CoV-2 [10]. The aim of this study was to investigate the value of lymphocyte count, IL-6 level and their combination in predicting the severity and prognosis of patients with SARS-CoV-2 pneumonia.

## Materials and methods

The procedures used for this study conformed to the standards of medical ethics. All subjects signed an informed consent form before peripheral venous blood was collected.

### Research subjects

A total of 109 patients who were diagnosed with SARS-CoV-2 pneumonia and received medical treatment at Nanjing First Hospital from December 2022 to January 2023 were enrolled.

#### Inclusion criteria

① Epidemiological history of COVID-19 (clustered onset or close contact with patients with confirmed COVID-19); ② pneumonia-related clinical symptoms and imaging manifestations (fever, respiratory symptoms or changes in viral pneumonia on chest CT); ③ corresponding aetiological evidence (positive nucleic acid test for SARS-CoV-2 via a nasopharyngeal swab or blood sample); and ④ peripheral blood lymphocytes lower than 1.0 × 10^9^/L for 5 d after admission.

#### Exclusion criteria

① Patients using immunosuppressive agents; ② patients with haematological diseases or autoimmune deficiency diseases that affect peripheral blood lymphocyte counts; and ③ patients with malignant tumours.

#### Diagnosis basis

Clinical classification was carried out using China's *Diagnosis and Treatment Protocol for Novel Coronavirus Pneumonia* (Trial Version 10).

COVID-19 was considered severe when any of the following criteria was met and could not be explained by reasons other than COVID-19: ① Shortness of breath, respiratory rate ≥ 30 times/min; ② resting state oxygen saturation ≤ 93% during inhalation; ③ arterial oxygen partial pressure (PaO_2_)/inhaled oxygen concentration (FiO_2_) ≤ 300 mmHg (1 mmHg = 0.133 kPa); PaO_2_/FiO_2_ was corrected in high altitude areas (over 1000 m above sea level) using the following formula: PaO_2_/FiO_2_ × [760/atmospheric pressure (mmHg)]; and ④ progressively worsening clinical symptoms and lesion progression > 50% within 24–48 h, as determined via lung imaging.

COVID-19 was considered critical when any of the following conditions were met: ① respiratory failure and need for mechanical ventilation; ② shock; and ③ organ failure requiring intensive care unit (ICU) monitoring and treatment.

#### Study grouping

**Fig. 1:**
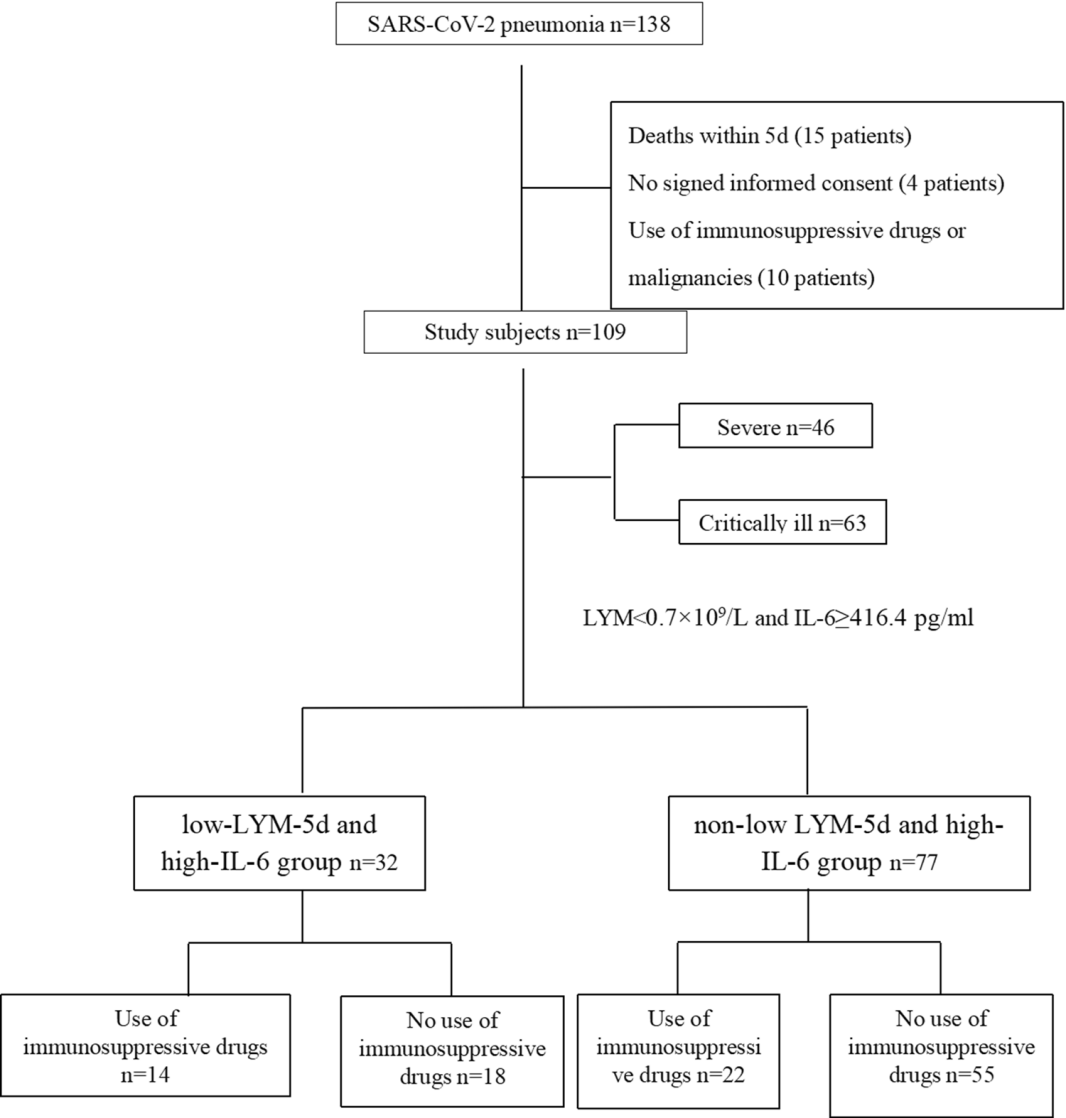
Flow chart of patient enrolment

The 109 patients included in the study were divided into severe (46 patients) and critically ill (63 patients) groups based on disease severity; the optimal cut-off values of IL-6 and LYM-5d, as determined using ROC curves, were used to regroup the patients. The patients were divided into a low LYM-5d and high-IL-6 group (32 patients) and a non-low LYM-5d and high-IL-6 group (77 patients) (Fig. 1).

### Method: clinical data

This was a prospective observational cohort study. Basic information (sex and age) of the patients was collected. Peripheral blood LYM, IL-6 and procalcitonin (PCT) values were measured from the day of inclusion to day 5 of the study. The hospitalization time, ICU stay and mechanical ventilation time of patients were recorded. The sequential organ failure assessment (SOFA) score was calculated to evaluate organ function. The proportion of patients with a secondary bacterial infection in the later stage was calculated. The relationship between treatment with the immunomodulatory agent thymosin (Beijing Saisheng Pharmaceutical Co., Ltd., State Drug Administration: H20065772; specification: 100 mg/pc; usage: 100 mg intravenous drip, once daily) and patient prognosis was investigated (Fig. 1). The primary endpoint was 28-day mortality, and the secondary endpoints were length of hospital stay, ICU stay, mechanical ventilation time and the effect of immunomodulatory agents on patient prognosis.

### Detection method of IL-6, PCT and LMY in peripheral venous blood

On the day of admission, 3 ml of peripheral venous blood was drawn from all patients into tubes containing EDTA (NanJing KeyGen Biotech Co., Ltd) as an anticoagulant. The IL-6 (machine model: Vazyme QD-S2000, Nanjing, China) and PCT (machine model: Vazyme QD-S2000, Nanjing, China) content and lymphocyte count (machine model: Mindray BC-7500CS, Shenzhen, China) were determined. Then, 3 ml of peripheral venous blood was drawn for 5 consecutive days to assess changes in the lymphocyte count.

### Statistical methods

SPSS 25 statistical software was used to analyse the data. To compare numerical variables, the Kolmogorov‒Smirnov normality test and Levene homogeneity of variance test were performed. Measurement data that conformed to a normal distribution are expressed as the mean ± standard deviation ($\overline{x}$ ± s) and were compared using the independent samples t test. Measurement data that did not conform to a normal distribution are expressed as the median (quartile) [M (QL, QU)] and were compared using the Mann‒Whitney U test. Count data are expressed as the number of patients and were compared using the χ^2^ test. Receiver operating characteristic (ROC) curves were plotted to analyse the value of the indexes in predicting disease severity. The Kaplan‒Meier method was used to analyse the survival time of patients, and a survival curve was plotted. Taking α = 0.05, *P* < 0.05 was considered statistically significant; all tests were two-sided.

## Results

### Comparison of the general data of patients

The 109 patients in this study were divided into a severe group (46 cases) and a critically ill group (63 cases) based on disease severity. There were 30 males and 16 females in the severe group (71 ± 17 years of age) and 46 males and 17 females in the critically ill group (78 ± 8 years of age). In the severe group and the critically ill group, 21 patients (45.7%) and 44 patients (69.8%), respectively, had hypertension; 8 patients (17.4%) and 24 patients (38.1%), respectively, had diabetes mellitus; and 6 patients (13.0%) and 23 patients (36.5%), respectively, had cerebrovascular disease. The proportion of patients with combined underlying disease was higher in the critically ill group than in the severe group. The SOFA score for the critically ill group was significantly higher than that of the severe group (t = 24.269, *P* < 0.001). LYM-5d, IL-6 and PCT at admission were significantly higher in the critically ill group than in the severe group (t/Z values, 4.515, 4.000, and 4.456, all *P* < 0.05). However, there was no significant difference between the two groups in the lymphocyte count on the first day (LYM-1d) (t = 1.400, *P* > 0.05) (Table 1).

**Table 1:** Comparison of the general data of patients

Item	Severe group (n = 46)	Critically ill group (n = 63)	*χ*^*2*^* / t/Z* value	*p* value
Sex (male, %)	30 (65.2)	46 (73.0)	0.766	0.381
Age (years, $\overline{x}$ ± s)	70.9 ± 16.9	78.3 ± 8.7	2.982	0.004^*^
*Underlying disease*				
Hypertension (cases, %)	21 (45.7)	44 (69.8)	6.462	0.011^*^
Diabetes (cases, %)	8 (17.4)	24 (38.1)	5.495	0.019^*^
Coronary heart disease (cases, %)	8 (17.4)	17 (27.0)	1.384	0.239
Cerebrovascular disease (cases, %)	6 (13.0)	23 (36.5)	7.496	0.006^*^
Kidney disease (cases, %)	3 (6.5)	8 (12.7)	1.118	0.290
SOFA score (points, $\overline{x}$ ± s)	1.9 ± 1.5	5.4 ± 3.0	24.269	< 0.001^**^
LYM-1d (× 10^9^/L, $\overline{x}$ ± s)	0.8 ± 0.4	0.7 ± 0.3	1.400	0.164
LYM-5d (× 10^9^/L, $\overline{x}$ ± s)	1.0 ± 0.4	0.6 ± 0.4	4.515	< 0.001^**^
IL-6〔pg/ml, M (QL, QU)〕	288.4 (191.4, 412.9)	513.0 (288.2, 857.4)	4.000	< 0.001^**^
PCT [ng/ml, M (QL, QU)]	0.1 (0.05, 0.2)	0.4 (0.1, 3.2)	4.456	< 0.001^**^

*LYM-1d* Lymphocyte count on the day of inclusion in the study, *LYM-5d* Lymphocyte count on the fifth day of inclusion in the study, *IL-6* Interleukin-6, *PCT* procalcitonin

*P < 0.05, **P < 0.001

### Comparison of the value of LYM-5d, IL-6 and LYM-5d + IL-6 for predicting SARS-CoV-2 pneumonia severity

**Fig. 2:**
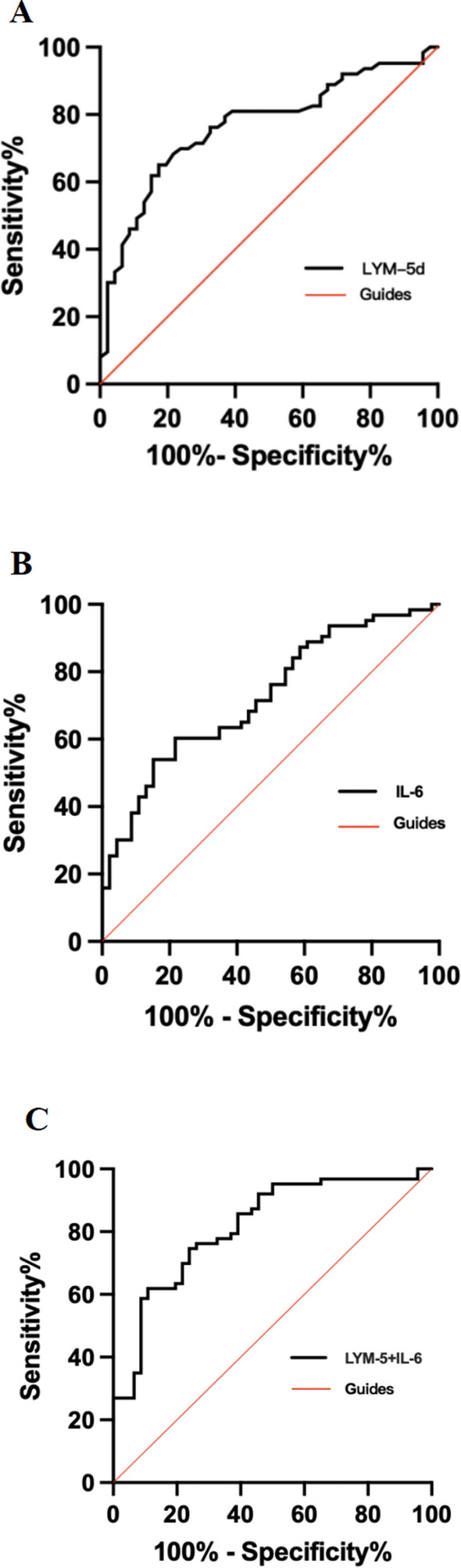
ROC curves for LYM-5d, IL-6 and LYM-5d + IL-6 and the prediction of SARS-CoV-2 pneumonia severity. **A** The ROC curve for LYM-5d and the prediction of SARS-CoV-2 pneumonia severity; **B** The ROC curve for IL-6 and the prediction of SARS-CoV-2 pneumonia severity; **C** The ROC curve for LYM-5d + IL-6 and the prediction of SARS-CoV-2 pneumonia severity

ROC curves were used to analyse the value of LYM-5d, IL-6 and LYM-5d + IL-6 for predicting SARS-CoV-2 pneumonia severity. The areas under the ROC curve (AUCs) for LYM-5d, IL-6 and LYM-5d + IL-6 were 0.766, 0.725, and 0.817, respectively (all P < 0.05), suggesting that all three indexes have value for predicting SARS-CoV-2 pneumonia severity; the 95% confidence intervals (95% CI) were 0.676 ~ 0.856, 0.631 ~ 0.819, and 0.737 ~ 0.897, respectively. The optimal cut-off values for LYM-5d and IL-6 were 0.7 × 10^9^/L and 416.4 pg/ml, respectively, the corresponding sensitivities were 65.1% and 60.3%, respectively, and the specificities were 82.6% and 78.3%, respectively. The sensitivity of LYM-5d + IL-6 for predicting disease severity was 61.9%, and the specificity was 89.1% (Table 2, Fig. 2A–C).

**Table 2:** Predictive value of IL-6, LYM-5d and their combination for SARS-CoV-2 pneumonia severity

Indicator	AUC	95% CI	*P* value	Sensitivity (%)	Specificity (%)	Optimal cut-off value
LYM-5d	0.766	0.676 ~ 0.856	< 0.001^**^	65.1	82.6	0.7
IL-6	0.725	0.631 ~ 0.819	< 0.001^**^	60.3	78.3	416.4
LYM-5d + IL-6	0.817	0.737 ~ 0.897	< 0.001^**^	61.9	89.1	–

*LYM-5d* Lymphocyte count on the 5th day of inclusion in the study, *IL-6* Interleukin-6

**P < 0.001

### The predictive value of different LYM-5d and IL-6 levels for patient prognosis

To further analyse the predictive value of LYM-5d combined with IL-6 for patient prognosis, the patients were regrouped based on the optimal cut-off values for LYM-5d and IL-6 and were divided into a low-LYM-5d and high-IL-6 group (LYM-5d < 0.7 × 10^9^/L and IL-6 ≥ 416.4 pg/ml), which included 32 patients, and a non–low-LYM-5d and high-IL-6 group (LYM-5 d ≥ 0.7 × 10^9^/L and IL-6 ≥ 416.4 pg/ml, LYM-5 d ≥ 0.7 × 10^9^/L and IL-6 < 416.4 pg/ml, and LYM-5 d < 0.7 × 10^9^/L and IL-6 < 416.4 pg/ml), which included 77 patients. The 28-day mortality was higher and the hospitalization time, ICU stay and mechanical ventilation time of patients in the low-LYM-5d and high-IL-6 group were longer than those of patients in the non–low-LYM-5d and high-IL-6 group; the incidence of secondary bacterial infections during the disease course was also higher, and the differences were statistically significant (Table 3).

**Table 3:** Comparison of the patient prognosis for different levels of IL-6 and LYM-5d

Item	Low-LYM-5d and high-IL-6 group (n = 32)	Non–low-LYM-5d and high-IL-6 group (n = 77)	χ^2^ / t/Z value	*P* value
28 d mortality (case, %)	23 (71.9)	23 (29.9)	16.352	< 0.001^**^
Hospitalization time (d, x ± s)	13.7 ± 6.3	8.4 ± 4.3	11.657	0.001^*^
ICU stay [d, M(QL, QU)]	9.0 (7.0, 11.5)	7.5 (4.0, 9.5)	2.113	0.002^*^
Mechanical ventilation time [d, M (QL, QU)]	8.0 (6.0, 10.0)	6.0 (3.3, 8.5)	2.553	0.002^*^
Secondary bacterial infection (cases, %)	24 (75.0)	32 (41.6)	10.120	0.001^*^

*P < 0.05, **P < 0.001

### Survival analysis of the low LYM-5d and high-IL-6 group and the non-low LYM-5d and high-IL-6 group

**Fig. 3:**
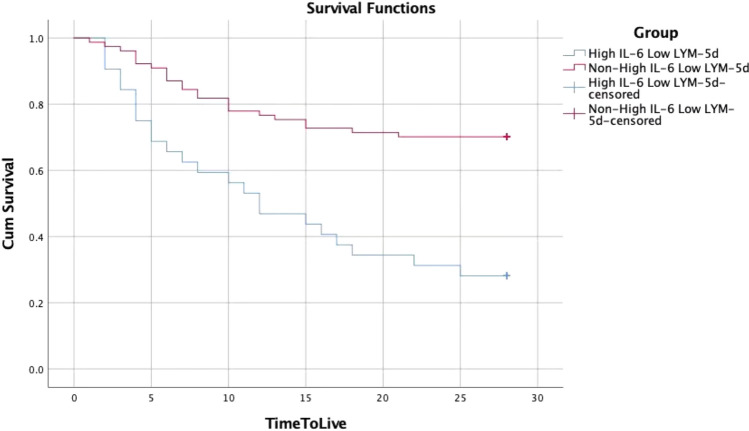
KM curve for the prognosis of patients with SARS-CoV-2 pneumonia in two groups with different LYM-5d and IL-6 levels

Kaplan‒Meier survival curve analysis of the low-LYM-5d and high-IL-6 group and the non-low LYM-5d and high-IL-6 group indicated that the median survival time of the former was 14.5 ± 1.8 d and that of the latter was 22.2 ± 1.1 d; the difference was statistically significant (Fig. 3).

### The effect of the immunomodulator thymosin on patient prognosis

In this study, intragroup statistical comparisons were performed based on the use or nonuse of the immunomodulator thymosin in the low-LYM-5d and high-IL-6 group and non-low LYM-5d and high- IL-6 group. In the low LYM-5d and high-IL-6 group, 14 patients used thymosin, 18 patients did not use thymosin, and 11 patients (78.6%) and 12 patients (66.7%) died on the 28th day, respectively; in the non-low LYM-5d and high-IL-6 group, 22 patients used thymosin, 55 patients did not use thymosin, and 5 patients (22.7%) and 18 patients (32.7%) died on the 28th day, respectively. There were no significant intragroup differences (Table 4).

**Table 4:** Comparison of the effects of immunomodulators on patient prognosis

Low-LYM-5d and high-IL-6 group		Non-low LYM-5d and high- IL-6 group	
Immunomodulators (n = 14)	No immunomodulators (n = 18)	Immunomodulators (n = 22)	No immunomodulators (n = 55)
11 (78.6)	12 (66.7)	5 (22.7)	18 (32.7)
0.552		0.750	
0.457		0.386	

## Discussion

Lymphocytes in patients with SARS-CoV-2 pneumonia can decrease to varying degrees, and the decrease in severe and critically ill patients is more substantial than that in mild and moderate patients [11]. The possible reasons for the lymphocyte decrease in COVID-19 patients are as follows: (1) the direct attack of SARS-CoV-2, resulting in lymphocyte apoptosis [12]; (2) after SARS-CoV-2 causes an inflammatory response in the body, the activated immune cells further produce cytokines, forming a cytokine storm and causing lymphocyte depletion [13]. Patients with persistently low lymphocyte counts (3–4 d) are likely to be in an abnormal immune state, and levels persistently lower than 1.0 × 10^9^/L are highly suggestive of immunosuppression [8]. Therefore, lymphocyte counts can be used as a rapid screening index for immune dysfunction in patients [3]. Lymphopenia has been shown to be associated with a poor prognosis for various diseases, such as COVID-19, sepsis and cancer [14]. A study focused on the trajectory of lymphocyte count changes, demonstrating a significant increase in mortality in patients with a five-day-long decrease in lymphocytes [15].

In this study, patients were divided into two groups, severe and critically ill, using China's *Diagnosis and Treatment Protocol for Novel Coronavirus Pneumonia* (Trial Version 10). Their IL-6 levels at admission and lymphocyte counts for five consecutive days were analysed. For patients with IL-6 ≥ 416.4 pg/ml at admission and a lymphocyte count < 0.7 × 10^9^/L on the 5th day, the SOFA score was high; the length of the hospital stay, ICU stay, and mechanical ventilation time were significantly prolonged; the 28-day mortality rate was higher; and the proportion of patients with secondary bacterial infections during the disease course was also higher, suggesting that the disease condition was more critical and the prognosis was worse.

The mechanism of the increase in IL-6 in COVID-19 patients may be the release of IL-6 from respiratory epithelial cells, CD14 + CD16 + monocyte-macrophages and lymphocytes after infection [16, 17]. In COVID-19, SARS-CoV-2 enters the body and causes excessive immune cell activation, resulting in the production of numerous inflammatory factors and the formation of inflammatory cytokine storms, which in turn cause systemic inflammatory response syndrome, multiple organ failure and ARDS, mainly manifesting as high fever, dyspnoea, lymphopenia, and increased cytokines [18]. In this study, the level of IL-6 on the day of hospitalization was used as an indicator. The justification for the choice is that in the early stage of severe infection, numerous inflammatory factors, mainly IL-6, are released, leading to proinflammatory reactions. The level of IL-6 often peaks in the early stage, resulting in a cytokine storm. IL-6 is essential for innate and adaptive immunity and can be a marker for diagnosing sepsis [19]. However, recent studies have found that the IL-6 level in patients with COVID-19 is significantly lower than that in patients with sepsis, cytokine release syndrome, and hyperinflammatory ARDS. The mechanism of SARS-CoV-2-induced multiorgan failure cannot be fully explained by a cytokine storm [20]. A study on the diagnostic and prognostic value of cytokines in sepsis and septic shock revealed that IL-6 can differentiate sepsis from nonsepsis; moreover, in patients with septic shock, IL-6 levels were significantly higher than those in patients with sepsis, and IL-6 > 348.9 pg/ml was an independent risk factor for a poor prognosis in patients with septic shock [21]. In this study, the level of IL-6 was associated with disease severity and prognosis, with a cut-off value of 416.4 pg/ml. IL-6 levels are elevated in COVID-19 patients, peak in critically ill patients, and gradually increase with disease severity [22].

Currently, the most studied method for inhibiting IL-6 is IL-6 antagonists, such as tocilizumab, which attenuate the activity of IL-6 by competing with IL-6 for binding to IL-6R. In response to high levels of IL-6 in COVID-19 patients, many hospitals use IL-6 antagonists to reduce inflammatory responses. Some retrospective studies have found that IL-6 antagonists alone can effectively block inflammatory storms, thereby reducing organ damage and mortality [23, 24]. However, some prospective studies have not found a significant effect on mortality with tocilizumab alone [25, 26]. In a multicentre study, the use of IL-6 antagonists alone did not significantly reduce mortality, but the combined use of IL-6 antagonists with a high-dose steroid shock reduced mortality and improved patient outcomes [27]. More controlled clinical studies are needed to confirm the efficacy of IL-6 antagonists on the new coronavirus and whether the combination of corticosteroids can improve clinical outcomes.

Thymosin α1 is a polypeptide hormone secreted by thymic epithelial cells that can effectively increase the number of T cells, promote the differentiation and maturation of T cells, and reduce apoptosis [28]. Studies have found that thymosin α1 can prevent cytokine storms, effectively shorten the length of hospital stay and reduce mortality [29, 30]. However, Wang et al. found that for all critically ill COVID-19 patients, the mortality rate was higher among those treated with glucocorticoids, immunoglobulins, and thymosin α1 [31]. A multicentre retrospective study found no relationship between thymosin α1 use and reduced mortality in critically ill COVID-19 patients [32]. That finding is consistent with the results of this study, which showed that use of the immunomodulator thymosin had no significant effect on the 28-day mortality of patients regardless of IL-6 levels and peripheral blood lymphocyte counts, indicating that thymosin treatment did not improve the clinical outcomes of patients. The main reason may be related to the fact that the lymphocyte counts of the subjects in this study were lower than normal when they were admitted to the hospital and that immune dysfunction existed in the early stage; this hypothesis needs to be tested through controlled clinical trials. Many COVID-19 patients have different degrees of immune dysfunction and an imbalance between proinflammatory responses and anti-inflammatory responses. Due to the complex mechanism of immune regulation, the selection of thymosin for treatment requires further clinical research.

The joint detection of LYM-5d and IL-6 serves to dynamically monitor the level of lymphocytes and cytokines and the immune function and inflammatory response of patients, thus allowing the early treatment of cytokine storms, thereby reducing organ damage and mortality. For immunosuppressed patients, improving immune function, enhancing immunity, and improving prognosis are precise treatment approaches for improving the prognosis of patients with SARS-CoV-2 pneumonia.

There are some limitations in this study. First, the Omicron strain was the predominant SARS-CoV-2 variant. The main variants recently prevalent in China were the BA.5.2 and BF.7 sublineages of BA.5, but which sublineage was prevalent in the Nanjing area was unknown, as sequencing was not performed. Second, only 28-day mortality was studied; 90-day mortality and a possible poor prognosis over the long-term were not addressed. Third, although a prospective observational cohort study was used, this was a single-centre study with a small sample size. Finally, due to the small number of patients who used thymosin α1, the efficacy results may not be accurate.

## Conclusion

In summary, the results of this study showed that patients with IL-6 ≥ 416.4 pg/ml at admission and a continuous decline in the lymphocyte count to < 0.7 × 10^9^/L by the fifth day have a higher 28-day mortality rate and a more critical condition, with a worse prognosis. Patients with low LYM-5d and high IL-6 levels had higher SOFA scores and longer hospitalization times, ICU stays and mechanical ventilation times, and a higher proportion of patients had secondary bacterial infections during the disease course, regardless of the IL-6 level and peripheral blood lymphocyte count. The use of thymosin immunomodulators had no significant effect on the 28-day mortality of patients, and thymosin treatment did not improve the clinical outcomes of patients.

## Data Availability

All relevant data are within the manuscript.
